# Supplementary material for: Cloud BioLinux: pre-configured and on-demand bioinformatics computing for the genomics community
Source: BMC Bioinformatics. 2012 Mar 19;13:42. doi: 10.1186/1471-2105-13-42 (PMC3372431; doi:10.1186/1471-2105-13-42)
Supplement: Additional file 1 — Supplementary 1 Cloud BioLinux software documentation in the form of a mini, self-contained website. Users need to download and uncompress the .zip file, and open through a web browser the "index.html" file available on the main directory. (ZIP 1823 kb). [file 1471-2105-13-42-S1.ZIP › Cloud-BioLinux-Package-Documentation/docs/xsplits.html]

Bio-Linux Software Documentation Pages

Back to search form

## xsplits

|  |  |
| --- | --- |
| Name | xsplits |
| Description | **xplits** is part of the splitstree package. Splitstree is used for analysing and visualising evolutionary data.  **xsplits** provides a menu driven graphical user interface for splitstree on unix and linux platforms.  See also the splits program, which is a command line program from the splitstree package.  **References:**  Huson, D.H. SplitsTree: analyzing and visualizing evolutionary data. Bioinformatics. 1998;14(1):68-73. [Entrez] |
| Homepage | http://bibiserv.techfak.uni-bielefeld.de/splits/ |
| Remote Documentation | http://bibiserv.techfak.uni-bielefeld.de/splits/manual.html |
